# Identification of Novel Type III Effectors Using Latent Dirichlet Allocation

**DOI:** 10.1155/2012/696190

**Published:** 2012-09-02

**Authors:** Yang Yang

**Affiliations:** Department of Computer Science and Engineering, Information Engineering College, Shanghai Maritime University, 1550 Haigang Avenue, Shanghai 201306, China

## Abstract

Among the six secretion systems identified in Gram-negative bacteria, the type III secretion system (T3SS) plays important roles in the disease development of pathogens. T3SS has attracted a great deal of research interests. However, the secretion mechanism has not been fully understood yet. Especially, the identification of effectors (secreted proteins) is an important and challenging task. This paper adopts machine learning methods to identify type III secreted effectors (T3SEs). We extract features from amino acid sequences and conduct feature reduction based on latent semantic information by using latent Dirichlet allocation model. The experimental results on *Pseudomonas syringae* data set demonstrate the good performance of the new methods.

## 1. Introduction

Secretion is an essential mechanism for bacterial adaptation and survival in their surrounding environment. The secretion process transports effector molecules from the interior of a bacterial cell to its exterior. Up to now, researchers have discovered six types of secretion systems. The type III secretion system is one of the most complex ones, which allows bacteria to deliver virulence effectors across eukaryotic cellular membranes [1].

In recent years, significant progress has been made in our understanding of the structural components of T3SS, including a needle-like component and bases embedded in the inner and outer bacterial membranes [2]. However, the details of the secretion mechanism and defined signals remain unknown. Identification of the effectors secreted by the T3SS (called type III secreted effectors, T3SEs) is very important to the T3SS study. They are believed to have some unique characteristics that can be recognized by the secretion system and be delivered into host cells. These characteristics are hints to uncover the mechanism of T3SS and understand the role that each component plays in the secretion process.

The amino acid sequences of T3SEs have great sequence diversity through fast evolution, and many T3SEs have very few homologous proteins in the public databases. Therefore, it is notoriously challenging to recognize T3SEs. The plant pathogen *Pseudomonas syringae* has been a model for the research of type III effectors. Thus far, only several hundreds of T3SEs have been identified and confirmed from all the bacterial species, and a large portion of them are from *P. syringae* strains. It indicates that a vast majority of T3SEs remain unknown.

This study aims to develop a computational prediction system, which can help the biologists to obtain the effector candidates for wet-bench experimental confirmation. Generally, the computational tools for predicting T3SEs can be divided into two types: sequence-based and domain knowledge-based.

The sequence-based methods usually attempt to extract discriminant subsequence features from amino acid sequences or nucleotide sequences and perform prediction based on these features. The features extracted from amino acid sequences include amino acid composition, *K*-mer frequencies [3, 4], and position-specific features [5]. As for the nucleotide sequences, genes encoding the T3SS apparatus and T3SEs usually have a conserved regulatory motif in their promoters [6]. Another sequence-based method, homology search using known effectors [3], is also often used, but it cannot identify novel effectors. The domain-knowledge-based methods include identifying genes in vicinity to chaperone homologues [7], predicting instability of N-terminus and nonoptimal codon usage [8], and using protein secondary structure and solvent accessibility information [9]. The domain knowledge is not as available as sequence data and usually obtained by computational approaches, which lowers the prediction accuracy.

This paper adopts machine learning methods to predict type III secreted effectors (T3SEs). The features are extracted from amino acid sequences. Researchers have detected amino acid composition biases in T3SEs, especially in the N-termini. For example, Guttman et al. [10] reported that the first 50 amino acids of *P. syringae* effectors have a high proportion of Ser and a low proportion of Asp residues. It should be noted that these observations only reveal some statistical biases instead of specific signals/features. Moreover, many effectors do not fulfill these requirements. In this paper, we regard the protein sequences as a kind of biological language and the *K*-mers as words. The word frequencies compose the feature vectors. In order to condense the feature space and improve the prediction accuracy, we propose two feature reduction methods. Both of them utilize latent semantic information in the latent Dirichlet allocation model [11].

We have examined the prediction accuracies of these two methods and compared them with four other methods, including dimer frequency, trimer frequency, and feature selection using frequency as well as *tf*-*idf* value. The methods were tested on the *Pseudomonas syringae* data set through fivefold cross-validation. The experimental results demonstrate the effectiveness of the proposed methods.

## 2. Methods

 Protein sequences are consecutive amino acid residues, which can be regarded as text strings with an alphabet *𝒜* of size |*𝒜* | = 20. The amino acid composition and *K*-mer (subsequence with length of *K*) frequency can be used as features for the protein sequence classification. The amino acid composition does not consider the order of amino acids, while *K*-mers retain some sequence order information, thus the latter method is usually adopted. However, the dimensionality of *K*-mer feature space grows exponentially as *K* increases. The prediction based on the full *K*-mer feature space without any dimension reduction would be computationally intractable. In fact, lots of *K*-mers are irrelevant to the prediction. For example, the *K*-mers appear only once or very few times.

In this paper, we propose two feature reduction methods based on latent Dirichlet allocation (LDA) model [11]. These two methods utilize the latent semantic information in different ways. One is to convert the original *K*-mer space to topic space, and the other is to use topic information to select informative *K*-mers for prediction. These two methods are introduced in Sections 2.2 and 2.3, respectively.

### 2.1. Latent Dirichlet Allocation

Latent Dirichlet allocation (LDA), the most common topic model currently in use, has been widely applied in natural language processing, image classification, social network analysis, and so forth [12, 13]. In LDA model, each document can be viewed as a mixture of various topics and that each word's creation is attributable to one of the document's topics.

**Figure 1:**
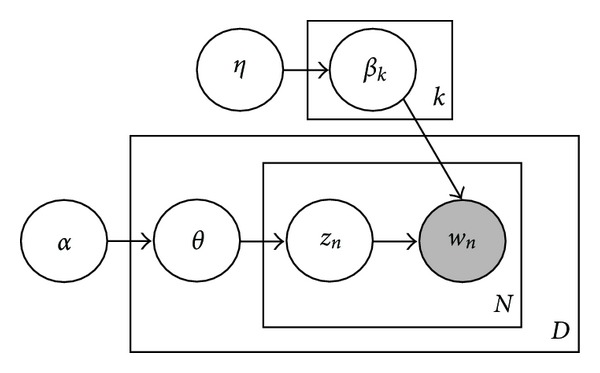
An LDA model.

Figure 1 shows a graphical model representation of LDA. (Here we consider the smoothed LDA.) The square frames represent replicates. There are *D* documents in the corpus, *N* words, and *K* topics. In this LDA model, a document is generated as the following steps.

Draw *θ* from the Dirichlet prior:

(1) $$\begin{array}{c} \theta \sim \text{Dir}\left(\alpha \right). \end{array}$$

For each word *w*_*n*_, pick a topic *z*_*n*_ from multinomial(*θ*), and then pick *w*_*n*_ from *p*(*w*_*n*_ | *z*_*n*_, *β*), which is a multinomial probability conditioned on the topic *z*_*n*_:

(2) $$\begin{array}{c} z_{n}\sim \text{Mult}\left(\theta \right)\\ w_{n}\sim p\left(w_{n}\;|\;z_{n},\beta \right). \end{array}$$

The likelihood of generating a corpus *𝒟* is defined in the following equation:

(3) p(𝒟 ∣ α,η)=∬∏k=1Kp(βk ∣ η)∏d=1Dp(θd ∣ α)×(∏n=1N∑znp(zn ∣ θ)p(wn ∣ zn,β))dθ dβ.

In this model, only *w*_*n*_ is fully observable. Inference of the hidden variables often adopts Gibbs sampling [14] or variational algorithms [15]. Since LDA is a generative model, with limited discriminative ability in classification tasks, we only use it for feature creation.

### 2.2. Prediction of T3SEs in the Topic Space

 In LDA model, each document is represented by a posterior Dirichlet over the topics. This is a much lower dimensional representation compared with using word frequency. Therefore, in this method, we create feature vectors by using the topic representation.

We regard protein sequences as text, and the *K*-mers are words. We would like to use LDA model to catch the latent topic information. Since the LDA model cannot be used directly on the protein sequences, we need first convert the protein sequences to a kind of biological language, whose words are the *K*-mers. Similar to Chinese sentences, we segment the amino acid sequences to nonoverlapping *K*-mers without spaces between words. After that, the LDA model can be applied on the sequences.

All the natural languages have predefined dictionaries. However, protein sequences are written in an unknown language to us at the present state, whose words are not delineated. Any combination of letters with arbitrary length may be a word. So we first need to build a dictionary, which is the basis of segmentation. Therefore, the whole process of this method consists of three steps: (1) construct a dictionary, that is, word set; (2) segment the protein sequences by matching the words in the dictionary, that is, *K*-mers; (3) run LDA model on the segmented sequences and create feature vectors.

We have tried two measures to determine the words to be included in the dictionary. One is word frequency, and the other is *tf*-*idf* value. They are defined in the following.

#### 2.2.1. Frequency

In natural language, words are generally the combinations of characters that frequently appear in the text. According to this observation, the amino acid substrings with high frequencies can be regarded as words, which should be segmented out and used as features. The unusual strings are noninformative for classification and have little influence on global performance. We record the appearance time for each *K*-mer in the training sequence set and preserve a predefined proportion of the most frequent *K*-mers.

#### 2.2.2. *tf*-*idf* Value

Considering that the frequency measure is apt to select the overrepresented words in the text, which may have little discriminant ability, we also use the *tf*-*idf* value. According to its definition in text categorization, *tf*-*idf* is calculated for a term in a single document. The value is in proportion to the number of occurrences of the term in the document, that is, the *tf* (term frequency) part; and in inverse proportion to the number of documents in the training set for which the term occurs at least once, that is, the *idf* (inverse document frequency) part.

Here we redefine it as the following equation. Let *w*_*t*,*s*_ be the *tf*-*idf* value for a *K*-mer *t* in sequence *s*, *f*_*t*,*s*_ be the frequency of *K*-mer *t* in sequence *s*, *N* be the size of the training set, and *n*_*t*_ be the number of sequences in which *t* appears:

(4) $$\begin{array}{c} w_{t,s}=f_{t,s}\times \log\frac{N}{n_{t}}. \end{array}$$

To avoid encountering unknown words, all 20 amino acids are included in the dictionary.

In the second step, we used the segmentation method proposed in [16]. This segmentation method has two criteria in searching the best way of segmentation. One is that the number of segments is the smallest. The other is that the product of the weights of the words segmented out is the biggest.

If the frequency measure is used in dictionary construction, the weight of word *t* is defined by frequency as follows:

(5) $$\begin{array}{c} w_{t}=\sum_{s=1}^{N}f_{t,s}. \end{array}$$

Or else, if the *tf*-*idf* measure is used, the weight of word is defined as the maximum value of *w*_*t*,*s*_, which is the *tf*-*idf* value for a *K*-mer *t* in sequence *s*:

(6) $$\begin{array}{c} w_{t}=\underset{s\in 𝒯}{\max\;}w_{t,s}, \end{array}$$

where *𝒯* denotes the whole data set. 

After segmentation, we run LDA model on the sequences. Then we obtain a sparse *D* × *T* matrix *A*, where *D* is the number of sequences and *T* is the number of topics. *A*(*d*, *j*) contains the number of times a word token in document *d* has been assigned to topic *j*. The row vectors are the feature vectors used in the classification. Here, we classify the protein sequences in the topic space instead of word space. Thus the dimensionality of the feature set can be greatly reduced because the number of topics are much less than that of words.

### 2.3. Prediction of T3SEs in the Reduced Word Space

In this method, the feature representation is totally different from the first method. Here we still use *K*-mer frequencies as features. Instead of using all the *K*-mers in the dictionary, we select informative ones according to the topic information.

The feature reduction process also consists of three steps. The first two steps are the same as in Section 2.2, while the third step needs certain strategies for selecting words.

Actually, the dictionary construction can be regarded as the initial screening procedure for word selection. The appearance times of the words in the dictionary can be recorded and compose the feature set. In the experiments, we examined the prediction accuracies of these two kinds of feature sets using frequency and *tf*-*idf* for word selection, respectively, and find that the frequency is better than *tf*-*idf* in this study (see the results shown in Table 2). Thus we conduct the third step based on the dictionary constructed by the criterion of word frequency.

Here we perform a further selection using topic information. We examine the number of times that words are assigned to topics and set a threshold *m*. If a word is not assigned to any topic at least *m* times, this word is discarded. In this way, we could remove the words which are either unusual words or not specific to any topic.

### 2.4. Complexity Analysis

 The computational time is mainly spent on sequence segmentation and LDA model. The segmentation algorithm [16] regards each amino acid as a check point. At each point, the algorithm conducts pruning by keeping only the optimal segmentation which has the least number of segments up to the current point, and search words by matching the subsequences next to the point with the words in the dictionary. Suppose that the dictionary size is *S*, the number of protein sequences in the data set is *D*, the average sequence length is *L*, and the maximum length of words is *M*, matching a word in the ordered dictionary has a cost of *O*(log⁡2*S*) by binary search. Thus, the computational complexity of the segmentation method is *O*(*DLM*log⁡2*S*) (*M* = 3 in the experiments). As for the LDA model, suppose there are *K* topics, the complexity is *O*(*DKL*) using Gibbs sampling technique for parameter estimation and inference. And in the second feature selection method, the complexity of selecting words is *O*(*KS*).

## 3. Results and Discussion

### 3.1. Data Set

 Since *Pseudomonas syringae* has been used as a model organism in the study of T3SEs, it has the most effectors that have been confirmed. Therefore, we collected data from this species. To our knowledge, there is a total of 283 effectors, have been confirmed, from *P. syringae* pv. tomato strain DC3000, *P. syringae* pv. syringae strain B728a, and *P. syringae* pv. phaseolicola strain 1448A. However, a large portion of them are homologs, that is, the sequence similarity is very high. This is because the homology-based search is still the major means to discover novel effectors. Considering that the redundancy of the data set would result in overestimation on the accuracy of the classifier, we eliminated the samples with sequence similarity over 60%. By removing the redundant sequences, we get a positive set of 108 samples.

The negative data set was extracted from the genome of *P. syringae* pv. tomato strain DC3000. We excluded all the proteins related to T3SS, as well as the hypothetical proteins. (Note that this set may still contain some unknown effectors.) And then we selected randomly from the remaining samples to constitute the negative set, since if we use all of them, the data set would be too much imbalanced. The numbers of the data sets are listed in Table 1.

### 3.2. Experimental Settings and Evaluation Criteria

 The classifier is built using the state-of-the-art supervised learning machinery, the SVM, which is widely used in bioinformatics. Our implementation of the SVM adopted LibSVM version 2.8 [17]. We considered polynomial, sigmoid, and RBF kernels for the SVM and observed that the RBF kernel has the best classification accuracy.

We used LDA model in the Matlab Topic Modeling Toolbox 1.4 [18]. As in LDA, the number of topics has great impact on its performance. The optimum number of topics was searched as described in Section 3.3. The other parameters used in the LDA model are set as follows: *β* = 0.01, *α* = 50/*T*, where *T* is the number of topics, and the number of iterations is 500. The threshold *m* is set to be 40 according to the statistics of word occurrence times.

Multiple measures were used to assess the performance of our proposed method, including sensitivity, specificity, and total accuracy (TA). The sensitivity and specificity can be defined in terms of the number of true positives (TPs), the number of false positives (FPs), the number of false negatives (FNs) and the number of true negatives (TNs) as follows. We define

(7) $$\begin{array}{c} \text{Sensitivity}=\frac{\text{TP}}{\text{TP}+\text{FN}}\\ \text{Specificity}=\frac{\text{TN}}{\text{TN}+\text{FP}}. \end{array}$$

These two measures examine the ability of the correct classification for positive and negative samples, respectively. TA is the ratio of the samples classified correctly compared to the total size of the data set, which is calculated as follows:

(8) $$\begin{array}{c} \text{TA}=\frac{\text{TP}+\text{TN}}{\text{TP}+\text{FP}+\text{TN}+\text{FN}}. \end{array}$$

Considering that the maximal secretion or translocation may require the first 100 amino acids [19–21], in our experiments, the first 100 amino acids were used.

### 3.3. Number of Topics

The number of topics is a key parameter in LDA model because it directly influences the performance of the model. The perplexity is frequently used to assess the performance of LDA models. It measures the performance of the model, which is defined by [11]:

(9) $$\begin{array}{c} \text{perplexity}\;\left(D_{\text{test}}\right)=\exp\left\{-\frac{\sum_{d=1}^{M}\log p\left(w_{d}\right)}{\sum_{d=1}^{M}N_{d}}\right\}. \end{array}$$

This measure decreases monotonically in the likelihood of the test data; thus lower values indicate better modeling performance.

**Figure 2:**
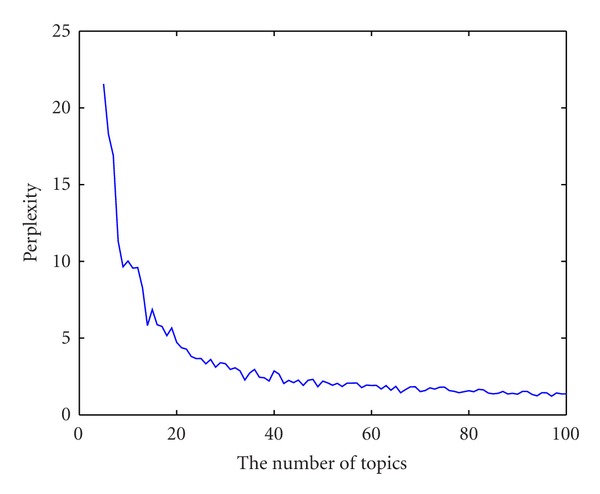
Perplexity under different numbers of topics.

We calculated the values of perplexity on a held-out dataset.  Figure 2 shows the perplexity plotted against the number of hidden topics, from 5 to 100. It can be observed that the perplexity decreases with an increasing number of topics. From 5 topics to 40 topics, the perplexity drops rapidly. When the number of topics is bigger than 40, the perplexity is almost constant. In our experiment, we set the number of topics to be 50.

### 3.4. Experimental Results

We have conducted a series of experiments to examine the performance of these two feature reduction methods and compared them with four other methods. Table 2 lists the number of dimension, total accuracy (TA), sensitivity, and specificity of these six methods. The method abbreviations and their corresponding description are in the following:

1. dimer: using all the dimers without feature reduction;
2. trimer: using all the trimers without feature reduction;
3. frequency: using the dictionary words selected by word frequency;
4. *tf*-*idf*: using the dictionary words selected by *tf*-*idf* value;
5. FRI: using topic information as features;
6. FRII: using the feature set which is based on (3) but further condensed by topic information.

From Table 2, we can find that all the six methods obtain total accuracies over 90%, which indicates that the amino acid patterns are competent for discriminating effectors and noneffectors.

In this study, long *K*-mers have little advantage for the classification. The dimer method has better performance than tri-mer method. Although the trimer method obtains a sensitivity of 100%, its total accuracy and specificity are much lower than other methods. That is because its false positive rate is very high. Since the prediction system aims to provide a reliable prediction result of effector candidates, the high false positive rate is not allowed.

Basically, all the new methods have satisfying performance. The feature selection methods using the dictionary words selected by frequency or *tf*-*idf* value achieve the best specificities and have overall better performance than the original dimer and trimer methods. It demonstrates that the strategy of dictionary construction and segmentation is successful in the protein sequence classification. The numbers of dimensions of these two methods are 220, including 20 amino acids, 150 dimers, and 50 trimers. The measure of frequency is better than *tf*-*idf* value, since the latter has a lower sensitivity. That may because the *tf*-*idf* value prefers to select some unusual words, which are not helpful for classification.

Obviously, the proposed feature reduction method I (FRI) has the smallest number of dimensions, but its accuracy is relatively low. FRII has 184 dimensions, including 20 single amino acids, 137 dimers, and 27 trimers. More trimers are discarded than dimers, because the frequencies of trimers are much lower and only a few of them can pass the criterion of word selection in Section 2.3. Actually, more trimers cannot improve the accuracy as we have mentioned before.

FRII achieves good results, even better than using all the dictionary words. The sensitivity of FRII is 2% higher than that of the frequency method, and the total accuracy and specificity are also comparable or better than other methods. These results indicate that although the topic space is not enough for the classification, the latent topic information is effective in selecting features.

## 4. Conclusions

 This paper focuses on the feature reduction methods for identifying proteins secreted via the type III secretion system using machine learning approaches. Our goal is to extract features from N-terminal amino acid sequences and use the classifier to discriminate the input feature vectors as secreted or nonsecreted proteins.

We have compared six methods including the *K*-mer methods without feature reduction and other methods with different feature reduction approaches. Computational experiments were conducted on *Pseudomonas syringae* data set. The cross-validation tests on the *P. syringae* data set show that our methods achieve high accuracies.

We observe that, while long *K*-mer features have little contribution in discriminating effectors and noneffectors, conducting feature reduction can improve the prediction accuracy. The methods using frequency and *tf*-*idf* value for word selection achieve better accuracies than *K*-mer methods, and the further feature selection using topic information can improve the performance and condense the feature space at the same time.

Thus far, a large portion of T3SEs in Gram-negative bacteria still remain unknown. The bioinformatics tools are of great importance. We believe that the new computational methods will contribute to the identification of novel type III secreted effectors and advance our understanding on TTSS.

As for the future work, the latent semantic information revealed by the topic models will be further investigated. LDA introduces a latent layer, which represents topic/subject in documents, or scene in images. For protein sequences, the latent layer could be secondary or spatial structure, function domain, or other biochemical properties. Since it is not as easy as images for proteins to visualize the sequences after running LDA, it is hard to define the specific corresponding concept of latent topic in the protein sequences. We will keep exploring the connection between biological characteristics and topics and incorporate other available information to discover the underlying mechanisms of the secretion system.

## Figures and Tables

**Table 1:** Data distribution.

Dataset	Number
Positive set	108
Negative set	760

Total	868

**Table 2:** Result comparison.

Method	Dimension	TA (%)	Sensitivity (%)	Specificity (%)
Dimer	400	94.2	91.4	94.5
Trimer	8000	90.4	100.0	90.2
Frequency	220	95.3	92.4	95.6
*tf-idf*	220	94.7	88.8	95.3
FRI	50	91.2	83.3	91.7
FRII	184	95.0	94.5	95.1
